# Aberrant innate immune sensing leads to the rapid progression of idiopathic pulmonary fibrosis

**DOI:** 10.1186/1755-1536-5-S1-S3

**Published:** 2012-06-06

**Authors:** Cory M Hogaboam, Glenda Trujillo, Fernando J Martinez

**Affiliations:** 1 Department of Pathology, University of Michigan Medical School, USA; 2 SUNY Stonybrook, NY, USA; 3 Pulmonary and Critical Care Medicine, Department of Internal Medicine, University of Michigan Medical School, USA

## Abstract

Novel approaches are needed to define subgroups of patients with Idiopathic pulmonary fibrosis (IPF) at risk for acute exacerbations and/or accelerated progression of this generally fatal disease. Progression of disease is an integral component of IPF with a median survival of 3 to 5 years. Conversely, a high degree of variability in disease progression has been reported among series. The characteristics of patients at risk of earlier death predominantly rely on baseline HRCT appearance, but this concept that has been challenged. Disparate physiological approaches have also been taken to identify patients at risk of mortality, with varying results. We hypothesized that the rapid decline in lung function in IPF may be a consequence of an abnormal host response to pathogen-associated molecular patterns (PAMPs), leading to aberrant activation in fibroblasts and fibrosis. Analysis of upper and lower lobe surgical lung biopsies (SLBs) indicated that TLR9, a hypomethylated CpG DNA receptor, is prominently expressed at the transcript and protein level, most notably in biopsies from rapidly progressive IPF patients. Surprisingly, fibroblasts appeared to be a major cellular source of TLR9 expression in IPF biopsies from this group of progressors. Further, CpG DNA promoted profibrotic cytokine and chemokine synthesis in isolated human IPF fibroblasts, most markedly again in cells from patients with the rapidly progressive IPF phenotype, in a TLR9-dependent manner. Finally, CpG DNA exacerbated fibrosis in an in vivo model initiated by the adoptive transfer of primary fibroblasts derived from patients who exhibited rapidly progressing fibrosis. Together, these data suggested that TLR9 activation via hypomethylated DNA might be an important mechanism in promoting fibrosis particularly in patients prone to rapidly progressing IPF.

## Introduction

IPF patients exhibit heterogeneous longitudinal behavior that is best defined by using a combination of physiological progression and clinical events. Identifying rapid progression over the first year of follow-up is clinically relevant given ongoing and planned therapeutic trials, which are of approximately one-year duration. Identifying which patients who are likely to experience rapid progression over such a time frame would provide key data in clinical decision-making and in the design of future therapeutic trials, providing an opportunity for personalized approaches to therapy. Unfortunately, baseline clinical, physiological and radiological differences, which identify these subgroups, have been difficult to identify.

Identifying biologically relevant differences between IPF patients that remain stable or show slow progression compared to those who rapidly progress is an active area of investigation but to date a detailed understanding of the gene expression pattern in IPF patients segregated into stable or rapidly progressive groups using standard methodology is lacking. Our studies to date suggest this is a promising avenue of investigation given that we have identified a host response gene, TLR9, which is uniquely upregulated in biopsies from rapidly progressing, but not slowly progressing, IPF patients relative to normal biopsies [1]. More importantly, human fibroblasts derived from lung biopsy material from rapidly progressing IPF patients respond to TLR9 activation via CpG-ODN [1].

## Results

### Physiology defines subgroups of IPF patients with varying disease progression

A high degree of heterogeneity in longitudinal physiological behavior is noted. In a group of 80 patients with IPF we examined the predictive ability of serial change in FVC over six or twelve months to predict subsequent survival [2]. At six months of initial follow-up 32% while at 12 months 54% of IPF patients exhibited a greater than 10% decrease in FVC. The predictive ability of a greater than 10% decrease in FVC at six months on subsequent mortality and the 12 month data were remarkably similar. In a larger study of IPF patients (n = 197), we examined the relationship of longitudinal change in FVC, DL_CO _or six minute walk performance stratified by baseline six-minute walk desaturation. An FVC decrease > 10% or a DL_CO _decrease of at least 15% was shown to strongly predict subsequent mortality or rapidly progressing IPF. Those patients who did not show these changes in lung function appeared to have a slower progressing IPF. Together, these data support that longitudinal physiological behavior can be used to segregate IPF patients into groups with differing long-term mortality, and physiological heterogeneity is a strong predictor of longitudinal disease course.

### Acute exacerbations contribute to mortality in IPF patients

**Figure 1:**
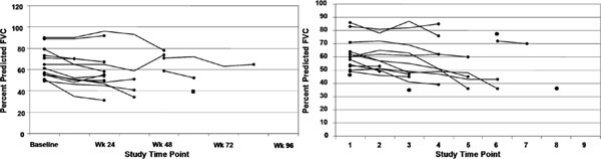
**A**. An inconsistent change in FVC is noted in IPF patients, who died acutely in the placebo arm of a large, randomized therapeutic trial. **B**. Serial FVC change in IPF patients, who died subacutely in the placebo arm of a large, randomized therapeutic trial.

In the placebo arm of a large therapeutic trial, we reported that 36 (21.4%) IPF patients died during 72 weeks of follow-up [3]. Death was considered by the primary investigators to be IPF-related in 89% of the cases. In 47% of these IPF-related deaths, investigators characterized disease progression as acute or abrupt suggesting that an acute exacerbation of disease was the reason for death. Figure 1 illustrates the longitudinal change in FVC among the IPF patients who died abruptly (Panel A) and those that died due to sub-acute deterioration (Panel B). A decrease in FVC was not consistently noted in patients who died acutely. These data highlight limitations of physiological deterioration in predicting mortality and suggested that additional endpoints are needed to define longitudinal phenotype behavior in IPF. The occurrence of acute exacerbations in IPF is an appropriate event-based component of a marker for disease progression.

To clarify the definition of acute exacerbations for clinical care and clinical trials a multinational, multi-investigator committee was convened with support from the NIH-sponsored Idiopathic Pulmonary Fibrosis Clinical Research Network. Table 1 shows the criteria used for a working diagnosis of an acute exacerbation. The totality of these data suggests that a phenotypic definition of rapid progression in IPF should include physiological change (FVC and/or DL_CO_), acute exacerbation or mortality (Table 2).

**Table 1:** Criteria suggested for defining an acute exacerbation of IPF

Previous or concurrent diagnosis of IPF
Unexplained worsening or development of dyspnea within 30 days
HRCT with new bilateral ground-glass abnormality and/or consolidation superimposed on a background reticular or honeycomb pattern consistent with UIP
No evidence of pulmonary infection by endotracheal aspirate or bronchoalveolar lavage
Exclusion of alternative causes, including the following:
Left heart failure
Pulmonary embolism
Identifiable cause of acute lung injury

**Table 2:** Methodology utilized to define slowly vs. rapidly progressive IPF

Physiological (over first year of follow-up)
Consistent FVC decrease > 10%
Consistent DL_CO _decrease > 15%
Event based (over first year of follow-up)
All cause mortality
Acute exacerbation

### TLR9 transcript expression is markedly increased in upper and lower lobe surgical lung biopsies from rapidly progressive but not slow IPF

In order to test the hypothesis that rapid progression of IPF might be a consequence of altered innate immune sensing and responsiveness to pathogen associated molecular patterns, we examined the relative levels of expression of TLR9 via quantitative PCR analysis in upper and lower lobe open lung biopsies obtained from patients with IPF compared with the histologically normal margins from resected lung tumors [1]. Normal tissues have low transcript expression for TLR9. Quantitative PCR analyses showed marked increases in the levels of expression of TLR9 in IPF SLBs relative to the levels of TLR9 expression detected in normal margins from resected lung tumors. TLR9 transcript expression in SLBs from rapidly progressive and slowly progressing IPF patients, and it appeared that TLR9 transcript expression relative to the normal biopsies was elevated in the rapidly progressive group versus slow IPF patients. Together, these data suggested that increased TLR9 expression might contribute to the rapidly progressive phenotype in IPF.

### TLR9 is expressed in the interstitial areas of rapidly progressive IPF SLBs

Molecular analysis through real time PCR showed that TLR9 expression was higher in IPF SLBs than in normal tissue; therefore to confirm these data and to localize the cellular source of pulmonary TLR9 mRNA expression, we performed immunohistochemistry on whole lung sections from IPF and normal margin surgical resection specimens. TLR9 staining was detected in SLBs from IPF patients. Of interest, TLR9 staining was localized not only in morphologically distinct immune cells but also in the alveolar epithelium (particularly in what appears morphologically to be type II cells) and in the interstitial areas of the IPF biopsies. TLR9 expression was present only in morphologically distinct immune cells but was not detected in interstitial areas of histologically normal lung tissue. Thus, these data confirmed that TLR9 protein was prominently expressed in IPF biopsy material, and provided evidence that the increase in TLR9 transcript expression in IPF was due to the expression of this receptor by cell types not normally positive for TLR9.

### TLR9-dependent, CpG ODN-induced cytokine and chemokine synthesis was noted in rapidly progressive IPF fibroblast cells but not slow IPF fibroblast cells

Based on the observation that TLR9 expression was remarkably enhanced in the interstitial areas of biopsies collected from patients affected by IPF, we have further investigated the expression of TLR9 protein in cultured IPF fibroblasts [4]. Using confocal microscopy, we detected high levels of TLR9 in the cytoplasm of untreated (i.e. media alone condition) fibroblasts from rapidly progressive IPF and the pattern of expression was homogeneous within the intracellular compartment. TLR9 was detected in slow IPF fibroblasts but the levels of expression were much lower than those detected in the rapidly progressive group. The TLR9 dependence of CpG ODN effects on human IPF fibroblasts was assessed using chloroquine. In these studies, the addition of CpG ODN to fibroblast cultures drove the expression of PDGF-BB, CXCL8, CXCL1, CCL5, CCL7, CCL11, and CCL2 in cultures of rapidly progressive fibroblasts but not in fibroblasts from slow IPF. Also, the presence of chloroquine in cultures of human fibroblasts reversed the CpG ODN effect in rapidly progressive fibroblasts. Importantly, there no evidence that this compound altered growth factor or chemokine generation by IPF fibroblasts due to toxic or non-specific effects when added alone. CCL2 in particular is a potent profibrotic mediator in the lung [5-7]. Together, these data suggest that CpG ODN drives chemokine synthesis in fibroblasts from rapidly progressive patients but not in fibroblasts from slow IPF patients. The stimulatory effect of CpG ODN in the former group of fibroblasts is TLR9-dependent.

### CpG ODN exacerbates the fibrotic response in a SCID model of IPF

To explore whether CpG ODN altered fibrosis in a model of IPF in SCID mice developed in our laboratory [1,8], we introduced CpG ODN by intranasal instillation into SCID mice, which had been intravenously injected with either normal fibroblast line or a rapidly progressive IPF human fibroblast line 35 days previously. Normal human fibroblasts injected into SCID mice did not evoke a fibrotic response in these mice as revealed by histological analysis 63 days later. The intranasal introduction of CpG ODN at day 35 alone did not alter the lung remodeling response. However, the introduction of IPF human fibroblasts alone into SCID mice markedly remodeled the SCID mouse lung 63 days later, and the fibrotic remodeling response in SCID mice intravenously challenged with IPF fibroblasts was dramatically enhanced with the CpG ODN treatment. Thus, the presence of CpG ODN markedly exacerbated the fibrotic response initiated by the introduction of human fibroblasts (a rapidly progressive line) into immunodeficient mice and lends further support to our hypothesis that altered responsiveness by fibroblasts to the presence of hypomethylated DNA can accelerate the course of fibrotic disease.

## Discussion and conclusions

It is becoming increasingly clear that IPF is a designation applied to a group of patients with heterogeneous longitudinal courses. Nearly half of all deaths are due to acute exacerbations superimposed on a chronic, more slowly progressive disease course. Our findings suggest that: 1) IPF patients exhibit variable longitudinal behavior both physiologically and clinically; 2) serial pulmonary function testing in combination with clinically relevant events (acute exacerbations and/or mortality) can be used to define clinically distinct subgroups of IPF patients; 3) TLR9 is strongly upregulated in IPF biopsies and expressed by IPF fibroblasts (most prominently in rapidly progressive patients); and 4) CpG ODN drives pro-fibrotic cytokines and chemokines in a TLR9-dependent manner strongly supporting the connection between host-environment interactions in pulmonary fibrosis.

## Competing interests

The authors declare that they have no competing interests.
